# Association of urinary bisphenol a concentration with type-2 diabetes mellitus

**DOI:** 10.1186/2052-336X-12-64

**Published:** 2014-03-13

**Authors:** Reza Ahmadkhaniha, Masoumeh Mansouri, Masud Yunesian, Kobra Omidfar, Maryam Zare Jeddi, Bagher Larijani, Alireza Mesdaghinia, Noushin Rastkari

**Affiliations:** 1 Department of Human Ecology, School of Public Health, Tehran University of Medical Sciences, Tehran, Iran; 2 Endocrinology and Metabolism Research Institute, Tehran University of Medical Sciences, Tehran, Iran; 3 Center for Air Pollution Research(CAPR), Institute for Environmental Research(IER), University of Medical Sciences, Tehran, Iran; 4 Department of Environmental Health Engineering, School of Public Health, Tehran University of Medical Sciences, Tehran, Iran; 5 Center for water qualities Research (CWQR), Institute for Environmental Research (IER), Tehran University of Medical Sciences, Tehran, Iran

**Keywords:** Urinary Bisphenol A (BPA), Diabetes mellitus, Solid-phase extraction

## Abstract

**Background:**

Bisphenol A as an endocrine-disrupting chemical is widely used chemical in the manufacture of polycarbonate plastics and epoxy resin and has become ubiquitous environmental contaminants. Human exposure to Bisphenol A is widespread and recent studies have been shown to be associated with a higher risk for self-reported adverse health outcomes that may lead to insulin resistance and the development of type-2 diabetes mellitus. In this context, we sought to confirm the association between Bisphenol A and diabetes in a community-based analysis of Bisphenol A urinary concentrations investigation in adult population of Iran.

**Methods:**

Regression models were adjusted for age, sex, Body Mass Index, serum triglyceride level and serum cholesterol level and serum creatinine concentration. Main outcomes were reported diagnoses of diabetes that defined according the latest American Diabetes Association guidelines.

**Results:**

The median age of the 239 participants was 51.65 years and 119 people had type-2 diabetes mellitus. Urinary Bisphenol A was categorized into two groups based on the median for Bisphenol A (≤0. 85 to >0.85 μg/L). The results of statistical analysis revealed a clear association between hypertension, and type 2 diabetes (*P* < 0.05). The multi variable-adjusted odds ratio for type-2 diabetes mellitus associated with the group 1 (referent), of urinary Bisphenol A was 57.6 (95% confidence interval: 21.10-157.05; *P*-value < 0.001). A positive correlation between HbA1c and urinary BPA concentration was observed (r = 0.63, *P* = 0.001).

**Conclusions:**

Urinary Bisphenol A levels are found to be associated with diabetes independent of traditional diabetes risk factors. Higher Bisphenol A exposure, reflected in higher urinary concentrations of Bisphenol A, is consistently associated with diabetes in the general adult population of the Iran. Studies to clarify the mechanisms of these associations are urgently needed.

## Background

In the modern world, humans are potentially exposed to a wide range of chemicals present in commonly used products. For the most of them, no information exists about the extent of the human exposure to these compounds, and the potential toxic effects of these chemicals are largely unknown. Bisphenol A (2,2′-bis[4hydroxyphenyl]propane; BPA) is among these compounds. BPA is used to manufacture polycarbonate plastic and epoxy resins, which are used in water containers and baby bottles, the resin linings of food and beverage cans, and for composites and sealants in dentistry and have become ubiquitous environmental contaminants [1,2].

BPA can be released into the environment during the production process and by leaching from the finished products [3]. Because of the widespread use of BPA, the potential for human exposure is high [4]. Data from the 2003–2004 National Health and Nutrition Examination Survey (NHANES) conducted by the Centers for Disease Control and Prevention (CDC) showed that 93% of individuals sampled among the american general population had detectable levels of BPA in their urine [5]. Results from many other researches also have shown that BPA exposure in different population groups [5-17]. Due to BPA endocrine disruptor properties, the potential metabolic effects of BPA are also of interest. Many different biological effects attributed to BPA have been described [18]. The mechanism explained for BPA actions is based on its binding to classic estrogenic receptors (ERα and ERβ), inducing estrogenic signals that change gene expression [19-24]. BPA has an affinity approximately1:2000 of that of 17β-E2 [21] and therefore it will activate estrogen receptors at concentrations within the micro molar level, which are lower than those commonly found in the environment [25].

Studies using rodent models have suggested that BPA can alter insulin biosynthesis and secretion in pancreatic b-cells, potentially through the over-activation of the estrogen receptor, ERα [19,26,27]. This may cause insulin resistance and the subsequent development of type-2diabetes mellitus (T2DM) [28]. Since orally administered BPA is rapidly and completely excreted, urine is considered most appropriate sample for assessment of BPA exposure [1]. Due to the previous animal studies, we hypothesized that higher urinary BPA concentrations would be associated with adverse effects, especially in the liver and in relation to insulin, T2DM, and obesity in humans. However, relatively few studies have done to examine the association between BPA and diabetes, and their results were not similar. Ning et al. and Kim et al. have reported that there is no association between urine BPA levels and diabetes [29,30], whereas Shankar et al. and Silver et al. have reported a significant positive association between urine BPA levels and diabetes [28,31]. In this context, we sought to confirm the association between BPA and T2DM in a community-based investigation in Iran.

## Methods

### Study design and participants

Two hundred and thirty nine male and female participated in this case–control study. Controls and cases were chosen from the Shariati Hospital outpatient clinic by simple randomized sampling (June until September 2012). Selected subjects were invited to a public health center for an interview and collection of a urine sample. Protocol of the study has been approved (Code: E-00126) by both the institutional review board and ethics committee of endocrinology and metabolic research institute (EMRI) of Tehran University of Medical Sciences. Written informed consent was obtained from the patient for the publication of this report and any accompanying images. The gathered data about participants’ included sex, age, blood pressure and current residence in face-to-face interviews. Height and weight were measured while subjects were wearing light clothing and no shoes. Body Mass Index (‘BMI’, measured weight in kilograms divided by the square of measured height in meters), categorized into: recommended weight (BMI ≤ 25.0 kg/m^2^) and overweight (BMI > 25.0 kg/m^2^). To measure urinary BPA concentration, morning spot urine sample was collected from each study participant. It has been reported in the literature that a single urinary level in one sample is sufficiently sensitive to demonstrate long-term exposure to BPA [32]. The patients’ ages ranged between 25 and 80 years. Exclusion criteria were creatinine >2 mg/dl, smoking, specific neurological disease (M.S, stroke, etc.) and consumption of sugared drinks in plastic bottles or canned food in two past weeks. The criteria for T2DM (case group) were based on self-reported and doctor-diagnosed type 2 diabetes according to the latest American Diabetes Association guideline (fasting plasma glucose level greater than 126 mg/dL, a glycosylated hemoglobin value greater than 6.5%) for more than one year (n = 119; age (mean ± SD) 56.64 ± 9.68 years; age range 28–79 years) and persons with fasting plasma glucose level less than 110 mg/dL, glycosylated hemoglobin value less than 6.5% and no history of diabetes were selected as control group (n = 120; age (mean ± SD) 46.71 ± 8.50 years; age range 29–74 years).

### Assessment of BPA concentrations

All the case and control participants supplied urine samples that were then analyzed for total BPA concentration. BPA was analyzed using solid-phase extraction coupled with gas chromatography–mass spectrometry after derivatization with BSTFA [33]. The limit of detection (LOD) and limit of quantification (LOQ) under the chromatographic conditions were determined at signal-to-noise ratios (S/N) of 3 and 6, respectively. The LOD was 0.1 μ/L and the LOQ was 0.2 μ/L. Individuals whose urinary concentrations fell below the LOD were assigned a value of LOD/2. A comprehensive quality control system, including reagent blanks, was used to ensure that samples were not contaminated during handling, storage, and analysis.

### Statistical analysis

Urinary BPA was categorized into two groups based on the median for BPA (≤0. 85 to >0.85 μg/L). We hypothesized that high BPA levels are associated with diabetes mellitus. The odds ratio (OR) [95% confidence interval (CI)] of diabetes for BPA was calculated by taking the lower BPA category as the referent and using multiple logistic regression models. We used three models. Model 1: unadjusted. Model 2: adjusted for age, sex, BMI and Hypertension, serum triglyceride level and serum cholesterol level and serum creatinine. Model 3: adjusted for significant covariates in model 2, And also, we used ROC Curve methods to calculate sensitivity and specificity of BPA for predicting diabetic or healthy samples.

## Results

The study involved 119 patients with T2DM and 120 non diabetic controls. The characteristics of the study population are provided in Table 1. Regression models were adjusted for potential confounders, including age; sex; BMI; blood pressure; serum creatinine; serum triglyceride level and serum cholesterol level. Multivariable logistic regression models were first elaborated to evaluate the crude association between the presence of T2DM and each individual risk factor. Age was significantly associated with increased risk of diabetes (P < 0.001). Mean age of diabetic persons was 10 years more than the control group. The results of statistical analysis demonstrate a clear association between hypertension, and T2DM (P < 0.05). The results showed that there was no significant association between sex and BMI and creatinine level with increasing risk of T2DM (P > 0.05) (Table 1).

**Table 1:** The univariate relationship analysis between type 2 diabetes mellitus and characteristics of the study population

**Characteristics**	**Diabetes**	**Control**	**P value**
	**119 (49.8)**	**120 (50.2)**	
Age (yr)	56.6 ± 9.7	46.7 ± 8.5	0.001
BMI (Kg/m^2^)	28.1 ± 4.4	27.4 ± 4.1	0.239
BPA (μg/L)	2.9 ± 1.8	0.5 ± 0.3	0.001
Hyperterntion			0.001
• Yes	42(17.5)	22(9.0)	
• No	77(32.2)	98(41.3)	
Triglyceride (mg/dL)	Triglyceride (mg/dL)	141.5 ± 74.0	0.08
Cholesterol (mg/dL)	Cholesterol (mg/dL)	192.1 ± 34.6	0.207
Creatinine (mg/dL)	Creatinine (mg/dL)	0.96 ± 0.18	0.182
Sex			
• Male	55 (46.2)	48 (40)	0.332
• Female	64 (53.8)	72 (60)	

Table 2 shows the results of three logistic regression models to examine the association between increasing levels of BPA and T2DM. Urinary BPA was categorized into two groups based on the median for BPA (≤0. 85 to >0.85 μg/L). The multi variable-adjusted odds ratio for type-2 diabetes mellitus associated with the group 1 (referent), of urinary BPA concentration was 57.56 (95% confidence interval: 21.10-157.05; *p*-value < 0.001).

**Table 2:** Logistic regression results to evaluate the association between BPA and type 2 diabetes mellitus in three various models

	**Predictor**		**Count**	**OR**	**C.I for OR**	***P *** **value**
Un adjusted model*	BPA	≤0.85(refrent)	119	-	-	-
		> 0.85	120	61.74	27.7-137.6	< 0.001
Adjusted for significant covariates in model 2	BPA	≤0.85(refrent)	67	-	-	-
		> 0.85	64	57.6	21.1-157.05	< 0.001
	Hyperterntion	Yes	44	4.9	1.97-12.5	0.001
		No(refrent)	87	-	-	-
	Age	-	-	1.14	1.07-1.02	0.049

*Full model (enter).

Positive associations were observed between increasing BPA levels and diabetes in all unadjusted, adjusted for age, sex, BMI and Hypertension, serum triglyceride level, serum cholesterol level and serum creatinine concentration and adjusted for significant covariates in model 2, (Table 2). Urinary BPA concentrations were higher in older adults, with no significant difference between male and female. Models evaluating trend in this association were also statistically significant (*P* < 0.05).

Finally, we assessed the correlations between urinary BPA concentration and levels of the hemoglobin A1c (HbA1c). Total HbA1c were (means ± S.D.) 6.64 ± 2.18 (range: 3.40–13.90). A positive correlation between HbA1c and urinary BPA concentration was observed (r = 0.63, *P* = 0.001).

**Figure 1:**
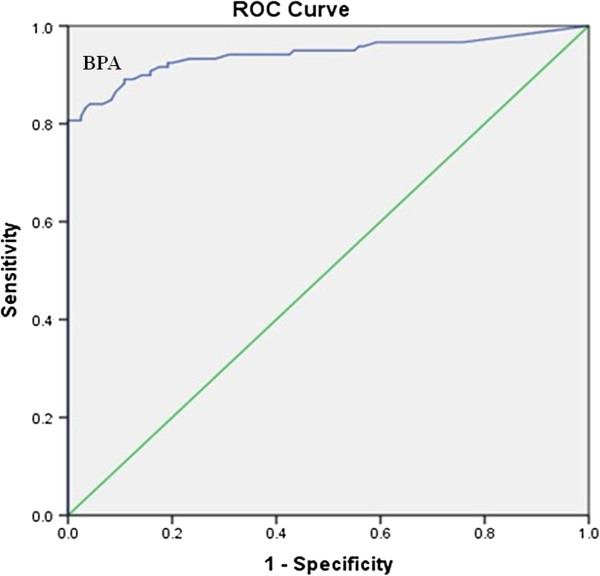
ROC carve analysis for bisphenol A (BPA).

Furthermore, the efficiency of urinary BPA concentration in discriminating T2DM patients were evaluated using ROC curve analyses was showed in Figure 1 (sens (%): 90, spec (%): 89).

## Discussion

With significant increases in prevalence, T2DM is considered an emerging pandemic in the world and is an important public health concern. Recently, attention has been paid to the potential contribution of BPA to the etiology of this disease.

Animal studies have suggested that BPA is an endocrine disruptor which might have various side effects on human health and may contribute to the development of T2DM. In addition, Alonso Magdalena et al. In a recent experiment demonstrated that low-level, chronic exposure to bisphenol A (BPA) induces insulin resistance, hyper insulinemia and glucose intolerance in adult mice. so animal studies suggest that an association between urinary BPA levels and diabetes mellitus may be plausible [19]. Therefore the aim of this study was to confirm the association between BPA and diabetes in a community-based investigation in Iran and this research add to the growing body of literature examining endocrine disruptor chemicals exposures and prevalence of T2DM in humans.

Our findings were in disagreement with Carwile and Michels results which showed that urinary BPA levels are associated with general and central obesity in the general adult population in the NHANES survey [34]. Therefore, future prospective studies are needed to confirm or disprove this finding.

In another study Lang et al. used NHANES (National Health and Nutrition Examination Survey) data from 2003/04 and found that higher BPA concentrations in urine were associated with diabetes and cardiovascular diagnoses, but not with other common diseases [35]. Melzer et al. then analyzed NHANES data from a subsequent survey, from 2005/06, and found that in those years, BPA levels were lower than they had been in 2003/04. The association between heart disease and BPA remained significant in 2005/06. The association between BPA and diabetes was significant in pooled data (2003–06), but did not reach significance in 2005/06 alone [36]. As a whole, the human studies sometimes find significant associations between BPA and diabetes, especially in the NHANES data from 2003/04, but not always, not in other years or in other surveys. Here, Significant and positive correlation were found between BPA concentrations and prevalence of T2DM. Our finding is in disagreement with some of previous studies which reported that there is no association between urine BPA levels and diabetes, whereas in agreement with Shankar et al. and Silver et al. who have showed a strong positive associations between the urinary concentrations of BPA and diabetes in adults. In these studies the observed association as found to be independent of confounding factors such as age, sex, BMI, urinary creatinine, serum triglyceride level, and serum cholesterol level [28,31]. Our study adds to the emerging evidence of the role of environmental exposure to BPA on endocrine-metabolic health in humans.

As regards recently, the International Expert Committee declared the use of the hemoglobin A1c (HbA1c), a measure of glycated hemoglobin in red blood cells, as an alternative method for the diagnosis of diabetes [37]. A positive correlation between HbA1c and urinary BPA levels was observed. Similar to our finding, Silver in 2011 reported a statistically significant association between urinary BPA and T2DM and HbA1c in the NHANES 2003/04 cycle in US Adults [28].

Our experiment has several major strengths. The advantage of our study over the some previous studies is that we defined diabetes in consistent with, the latest American Diabetes Association guidelines including fasting glucose and glycosylated hemoglobin levels in addition to self-reported diabetes [38]. We select HbA1c as the outcome for this study for diabetes indicator, HbA1c is relatively more stable comparing to other markers of glycemic indices, such as fasting blood glucose, and it provide appropriate indicant of average blood glucose over the previous six months [39]. Also, a continuous biomarker such as HbA1c would more representatives in undiagnosed disease, which could diminish the outcome misclassification of the control group. To our knowledge, this is the first study to investigate the association between BPA concentrations and the risk of T2DM among Iranian adults. The other strength of current study is that the LODs of our samples were relatively low compared with those of others, which may have led to higher detection rates.

## Conclusions

Consequently, we found that with a nationally representative sample of Iran adults, higher BPA levels were positively associated with diabetes mellitus independent of confounding factors. However this study has some limitations the current study is case–control in nature hence, making it impossible to draw a cause and effect in the observed associations. Additionally, because this study only examined BPA exposure among adults, it was impossible to investigate any effect of BPA exposure during critical growth periods, such as prenatally. Further study of this possible association is warranted. If the results of this research would be confirmed in future prospective studies, reducing BPA exposure may play a role in the prevention and reduce of diabetes mellitus incidence.

## Competing interests

The authors declare that they have no competing interests.

## Authors’ contributions

RA, MY and MM participated in the design of the study. MZJ did the analyses and MY interpreted the analyzed results. KO, AM and BL were advisors of the study. NR was the main investigator, supervised the work, drafted and revised the paper critically for important intellectual content and compiled the work in accordance to journal format. All authors have read and approved the final manuscript.
